# Editorial to the Special Issue: “Recent Advances in the Management of Chronic Pain”

**DOI:** 10.3390/ijerph20196875

**Published:** 2023-10-02

**Authors:** Marco Cascella

**Affiliations:** Department of Medicine, Surgery, and Dentistry, Unit of Anesthesiology, Intensive Care Medicine, and Pain Medicine, University of Salerno, Via Salvador Allende, 43, 84081 Baronissi, Italy; m.cascella@istitutotumori.na.it

Chronic pain is a complex biopsychosocial phenomenon with far-reaching implications, not only in terms of clinical care but also in the realms of social and economic impact [1,2]. It poses a formidable challenge for those involved in pain management, but the recent progress in this field is undeniably exciting [3]. Pieces of evidence suggest that the management of chronic pain requires the collaboration of multiple specialists dedicated to comprehensive patient care [4]. Therefore, recent advancements in chronic pain management span a wide range of modalities, including pharmacology [5], invasive techniques [6], psychological interventions [7], non-pharmacological approaches [8], and unconventional therapies [9]. Furthermore, it is becoming increasingly clear that simply measuring pain and providing relief is insufficient. It is imperative to integrate pain measurement, treatment efficacy, and assessments of the quality of life and functional recovery [10].

This collection of articles highlights the remarkable progress and insights in the field, offering valuable contributions to our understanding and treatment of chronic pain. Various specialists from both research and clinical backgrounds are converging on this issue, emphasizing the need for an interdisciplinary approach. Crucially, this collection of articles brings attention to the ongoing transformation in the field, emphasizing the importance of research, innovation, and evidence-based practice. It also serves as a call to action for policymakers to recognize the significance of chronic pain and invest in comprehensive strategies that encompass prevention, education, and access to effective treatments.

After the review process, eight papers were finally accepted for publication and inclusion in this Special Issue. The contributions are listed below:

As shown in Table 1, the contributions covered large geographical areas, although the majority of the articles (3,4,6,7,8) came from Italy. The collection included two observational studies, a qualitative investigation, a review article, and due papers addressing case reports on neuromodulation for the management of chronic noncancer pain (Table 1).

Contribution 1 addressed the topic of telemedicine. Telehealth strategies offer a robust solution for effectively managing pain [11,12,13] and this contribution regards the experiences of healthcare professionals (HCPs) in delivering virtual care to young individuals with chronic pain during the COVID-19 pandemic. Through qualitative interviews and quantitative satisfaction surveys, the study examines the adaptations, benefits, limitations, and evolving perspectives on remote care. The results indicate that HCPs are able to effectively provide diagnoses, recommendations, and care plans for pediatric chronic pain through virtual care. Notably, the study confirms the results of other research [14,15] and highlights the importance of understanding HCPs’ experiences to improve the delivery of telemedicine-based multidisciplinary strategies for children with chronic pain. The investigation also provides valuable insights for future guidelines in this area [16].

Contribution 2 focused on the important issue of chronic pain features and somatosensory impairment in stroke survivors. By examining the relationship between these two factors, the study provides interesting insights into the potential interplay and influence between pain and somatosensory function. The findings reveal that stroke survivors with altered somatosensory abilities are more likely to experience chronic pain, encompassing various sensory functions. This highlights the need to consider somatosensory impairment as a target for rehabilitation strategies aiming to improve pain-related outcomes in stroke survivors. The study contributes to our understanding of chronic pain in stroke populations and offers implications for enhancing their quality of life through targeted interventions.

Contribution 3 investigated the use of opioids for the management of non-cancer pain. It is a topic of considerable debate and controversy [17]. While these analgesics can provide effective pain relief, they also come with significant risks and potential adverse effects. In a retrospective analysis, the authors evaluated the effectiveness of an oxycodone–naloxone combination in reducing opioid consumption among patients with chronic low back pain (CLBP) caused by osteoarthritis. In particular, the study explored the hypothesis, supported by animal models, that combining ultra-low doses of opioid antagonists with opioid agonists can mitigate the development of tolerance. Fifty-three CLBP patients were treated with either oxycodone and prolonged-release naloxone (OXN) or oxycodone—controlled release. Throughout the two-year follow-up period, the study closely monitored the patients’ pain relief and opioid usage. The results revealed that individuals receiving OXN treatment required notably lower doses of oxycodone than those solely treated with oxycodone in order to achieve equivalent levels of pain relief. This suggests that proper pharmacological methods may hinder the development of opioid tolerance in long-term opioid treatment. While this study provides promising results, it is important to consider its limitations, including its retrospective design and relatively small sample size. Nevertheless, these findings can contribute to optimizing pain management strategies and improving outcomes for individuals with CLBP due to osteoarthritis, providing insights into alternative pharmacological approaches for managing chronic pain while minimizing the risks associated with prolonged opioid use [18].

Contribution 4 reviewed the most recent literature on the opioid epidemic phenomenon. Notably, it represents a serious public health crisis that has inflicted devastating consequences, resulting in a substantial rise in opioid-related overdoses, fatalities, and enduring health complications [19]. The authors presented an in-depth review of the key issues, challenges, and strategies surrounding opioid misuse in the context of chronic pain. By examining the factors contributing to opioid misuse and exploring preventive measures and intervention strategies, this article provides a comprehensive overview for healthcare professionals and policymakers to guide their efforts in combating the opioid crisis while ensuring effective pain management for patients. In this intricate context, the prudent utilization of opioids is imperative.

Contribution 5 highlighted several aspects of novel opioid dosing strategies and the potential advantages of buprenorphine micro dosing and cross-tapering in the management of chronic pain. These innovative approaches demonstrate promising outcomes in mitigating opioid-related risks while effectively addressing chronic pain, underscoring the significance of exploring alternative methods for opioid therapy [20].

Contribution 6 sheds light on a pressing concern. Despite the existence of Law 38 in Italy, which explicitly recognizes the right of individuals not to suffer, the authors emphasize that significant deficiencies persist in ensuring access to proper pain care. The authors highlight the multifaceted impact of chronic pain not only on individuals but also on society and healthcare systems. This calls for a closer examination of the current state of chronic pain management and the need for comprehensive efforts to address the gaps in providing adequate care for individuals living with chronic pain. In summary, an effective strategy for pain management must encompass the comprehensive treatment of the physical, emotional, and social aspects of the individual’s pain condition.

Contribution 7 underlined the advancements in technology and therapeutics. In two case reports, the authors illustrate the application of wireless peripheral nerve stimulation for managing chronic pain in the upper limb. This innovative treatment modality demonstrates the potential of advanced strategies in providing targeted and effective pain relief, offering new possibilities for patients who have experienced limited success with conventional therapies.

Contribution 8 described the safety and potential benefits of neurostimulation as a treatment option for managing CLBP in pregnancy. This case study addresses the unique considerations and challenges associated with pain management during pregnancy, providing valuable insights into the efficacy and safety of neurostimulation techniques for both the mother and the developing child [21].

In summary, the collection not only expands our understanding of this complex condition but also reinforces the imperative of collaboration, interdisciplinary approaches, and patient-centric care [22]. By leveraging the insights provided by these articles, healthcare professionals, researchers, and policymakers can make meaningful strides in improving outcomes and enhancing the quality of life for individuals living with chronic pain.

## Figures and Tables

**Table 1:** Summary of the published contributions in this Special Issue.

N# of Contribution	Country °	Topic(s)	Type of Article
1	Canada	Telemedicine	Qualitative Study
2	Australia	Chronic Pain	Observational Study
3	Italy	Chronic Low Back Pain and Opioids	Observational Study
4	Italy	Opioids	Review
5	USA	Opioids	Viewpoint
6	Italy	Chronic Pain Care	Review
7	Italy	Neurostimulation	Case report
8	Italy	Neurostimulation	Case report

Legend: ° Corresponding author.
